# Supplementary material for: A genome-wide association study identifies a locus associated with knee extension strength in older Japanese individuals
Source: Commun Biol. 2024 May 20;7:513. doi: 10.1038/s42003-024-06108-6 (PMC11106293; doi:10.1038/s42003-024-06108-6)
Supplement: Supplementary file 2 — Supplementary Information [file 42003_2024_6108_MOESM2_ESM.pdf]

Supplementary Information

A genome-wide association study identifies a locus associated with knee extension strength in older people

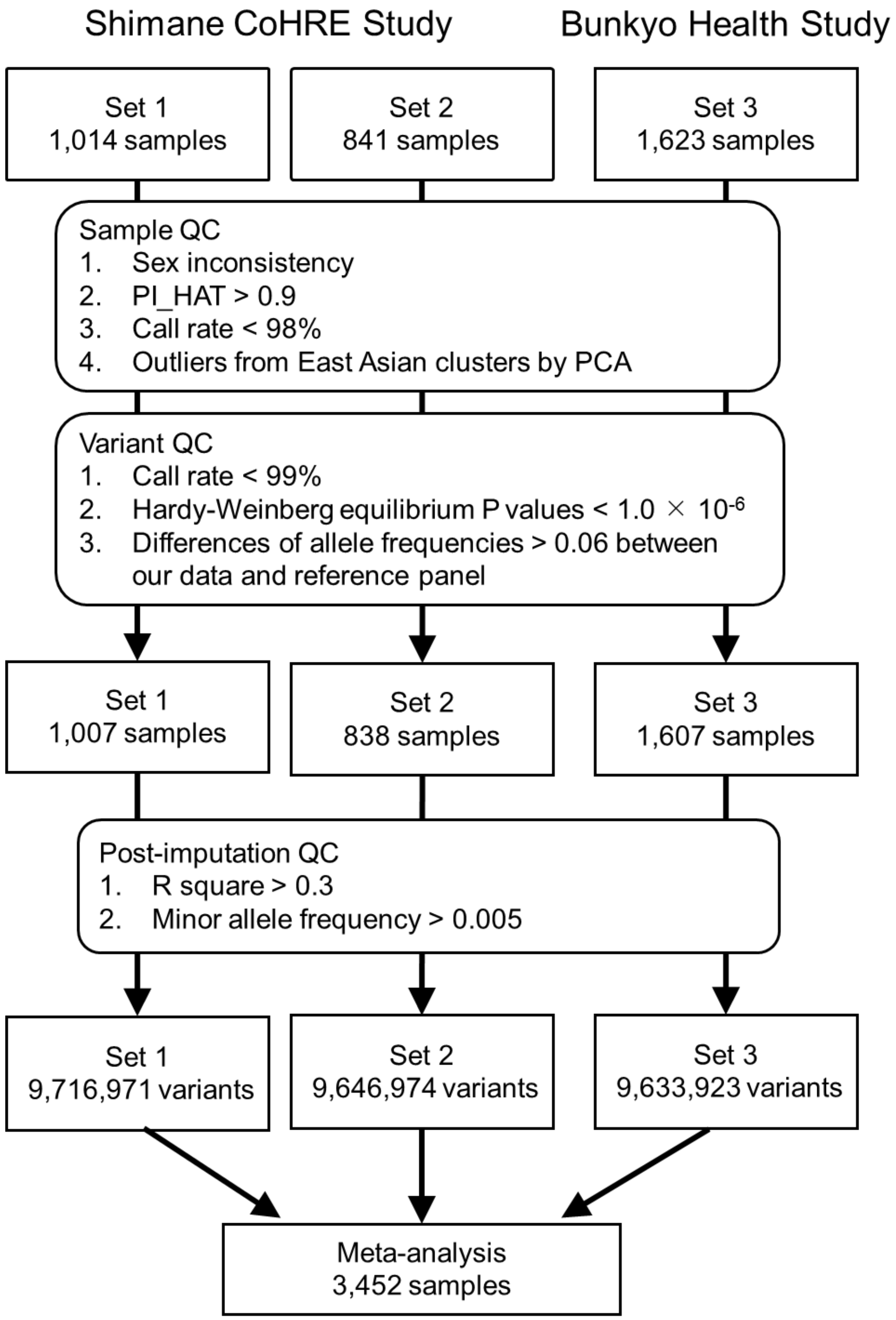

Supplementary Figure 1. Overview of the meta-analysis for the participants aged 60 years or older
Three genome-wide association studies (Set 1-3) from two cohort studies (Shimane CoHRE Study and Bunkyo Health Study) were analyzed. PCA, principal component analysis; QC, quality control.

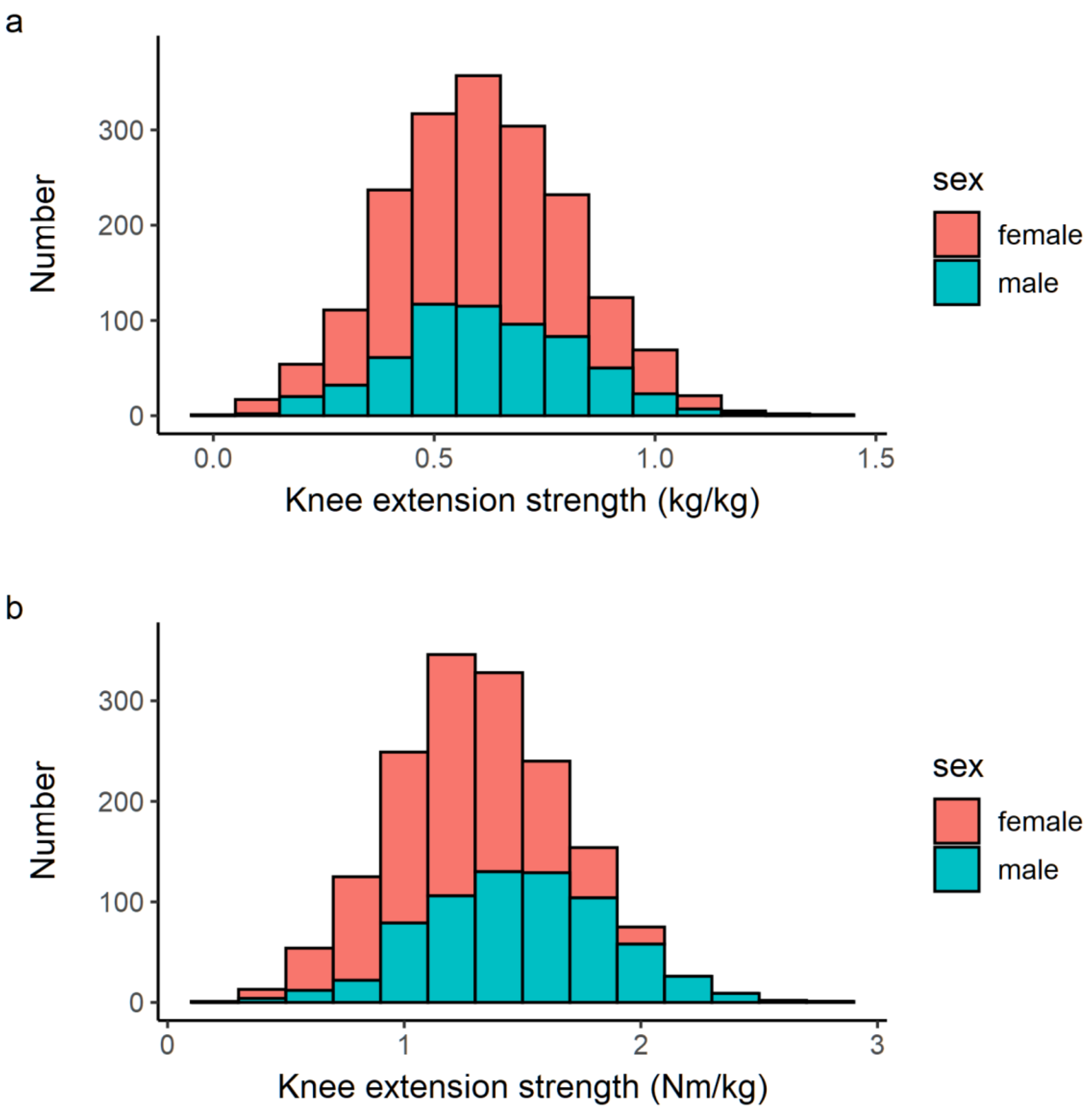

Supplementary Figure 2. Distribution of knee extension strength in participants aged 60 years or older
(a) Shimane CoHRE Study (Set 1 and Set 2), (b) Bunkyo Health Study (Set 3). Both studies show similar distribution.

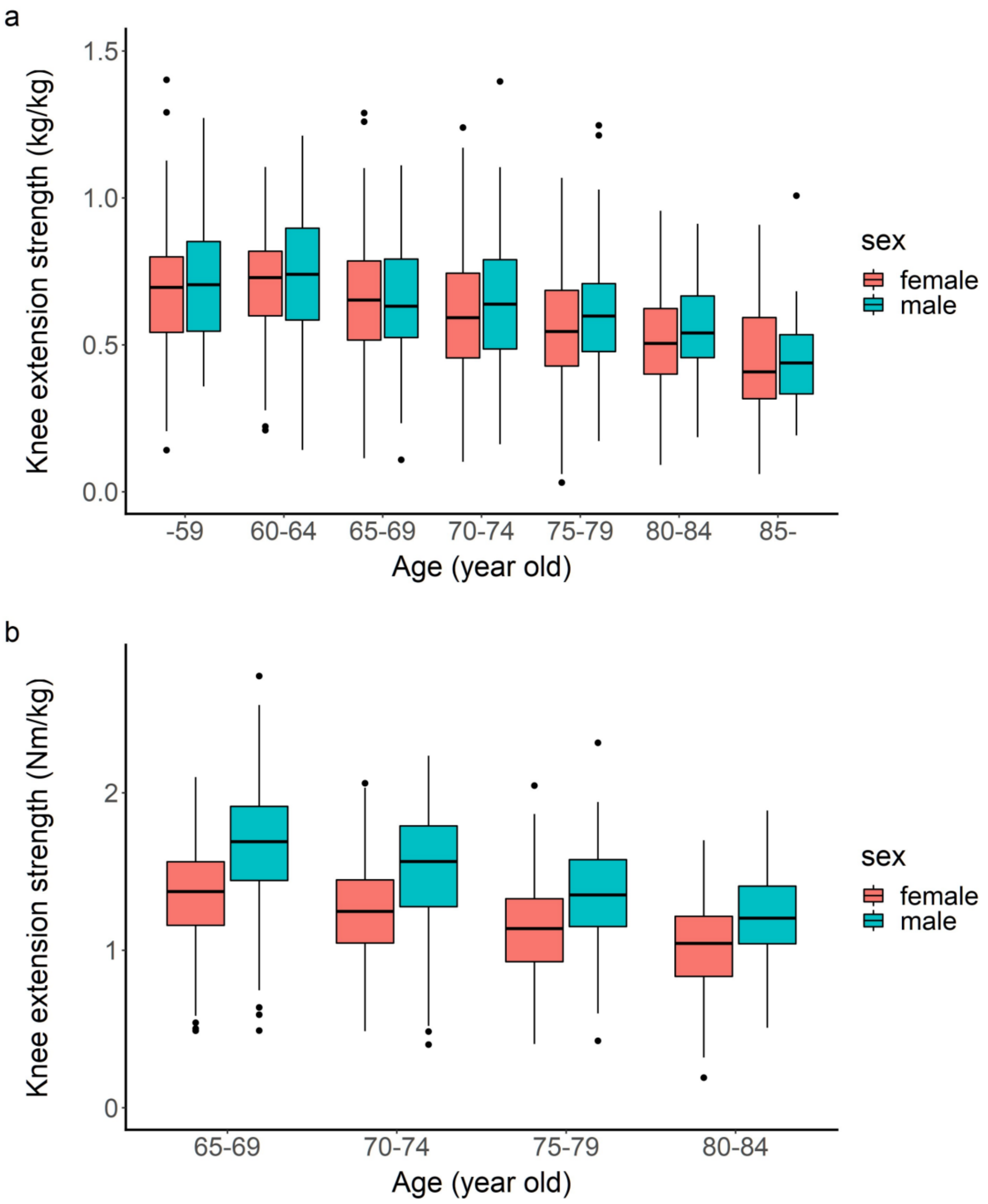

Supplementary Figure 3. Knee extension strength in age groups
(a) Shimane CoHRE Study. Set 1 and Set 2, (b) Bunkyo Health Study (Set 3). center line, median; box limits, upper and lower quartiles; whiskers, 1.5x interquartile range; points, outliers

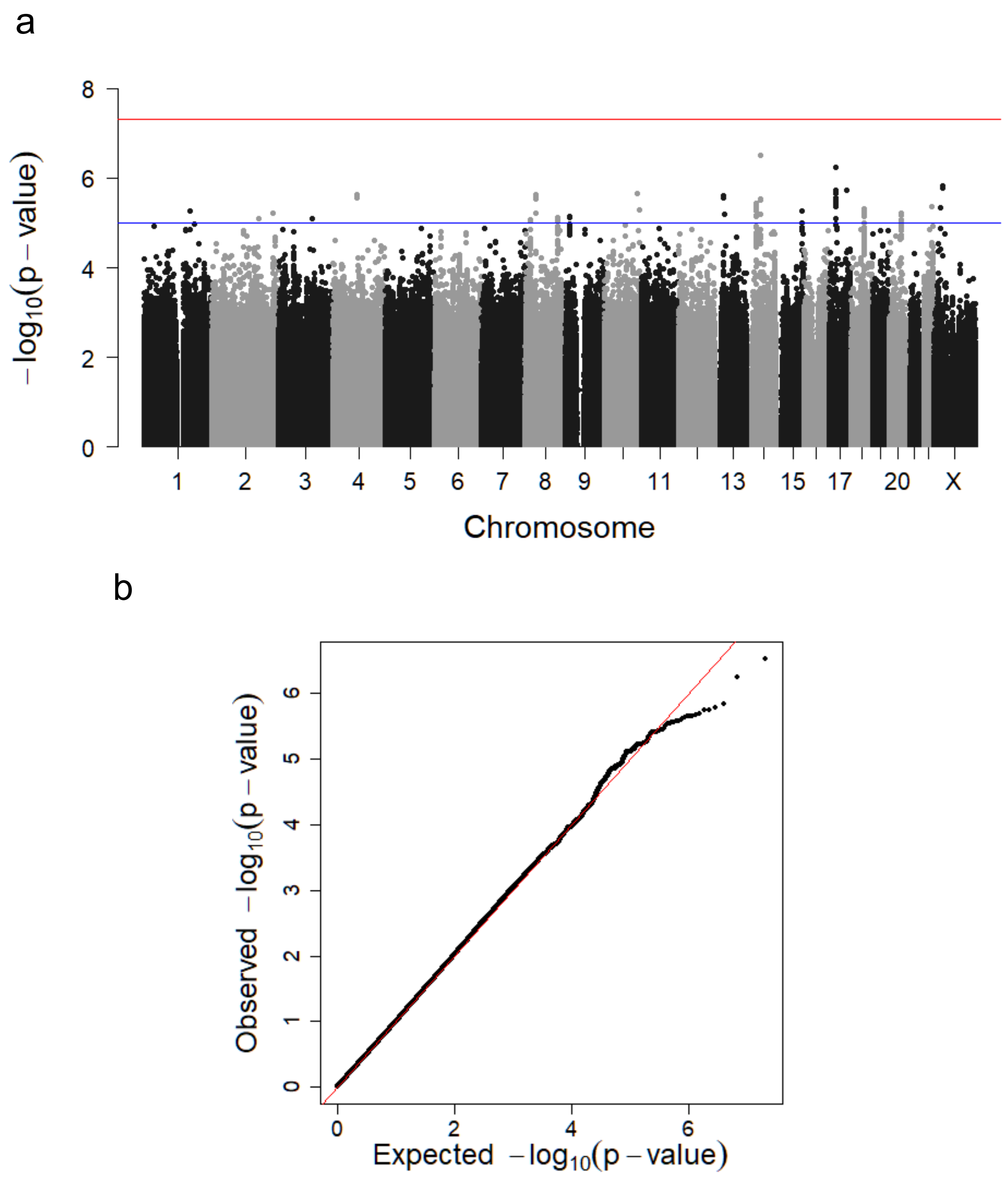

Supplementary Figure 4. A sub-analysis of the genome-wide association analysis of knee extension strength for 1,007 participants of Set 1 aged 60 years or older

(a) Manhattan plot. X-axis: chromosomal location. Y-axis: $-\log_{10}$ P value for each genetic variant. Horizontal red line: genome-wide significance ($p < 5 \times 10^{-8}$). Horizontal blue line: suggestive genome-wide significance ($p < 1 \times 10^{-5}$). (b) Q-Q plot for the analysis. The genomic inflation factor (λ_{GC}) was 1.00.

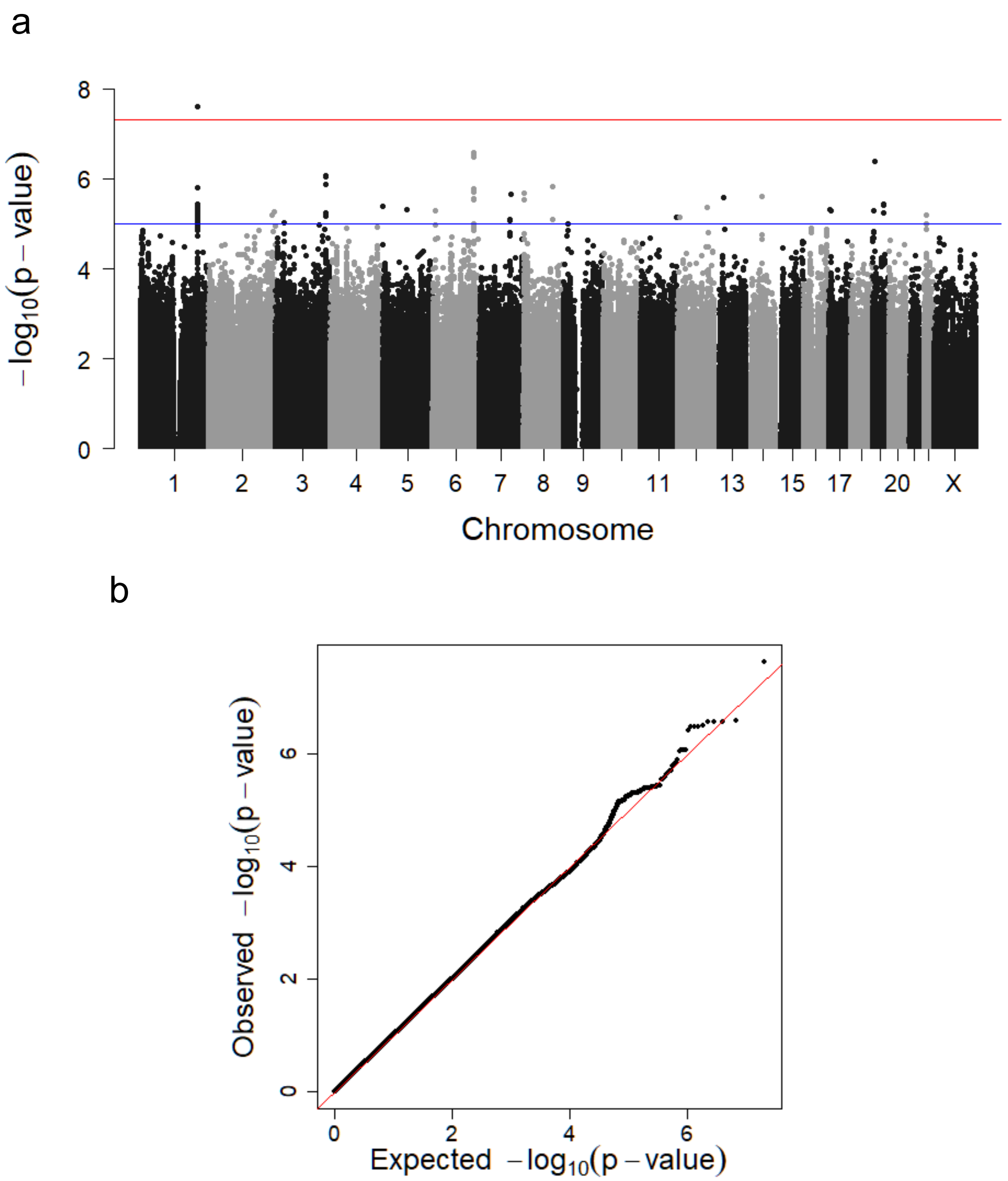

Supplementary Figure 5. A sub-analysis of the genome-wide association analysis of knee extension strength for 838 participants of Set 2 aged 60 years or older

(a) Manhattan plot. (b) Q-Q plot for the analysis. The genomic inflation factor (λ_{GC}) was 1.01.

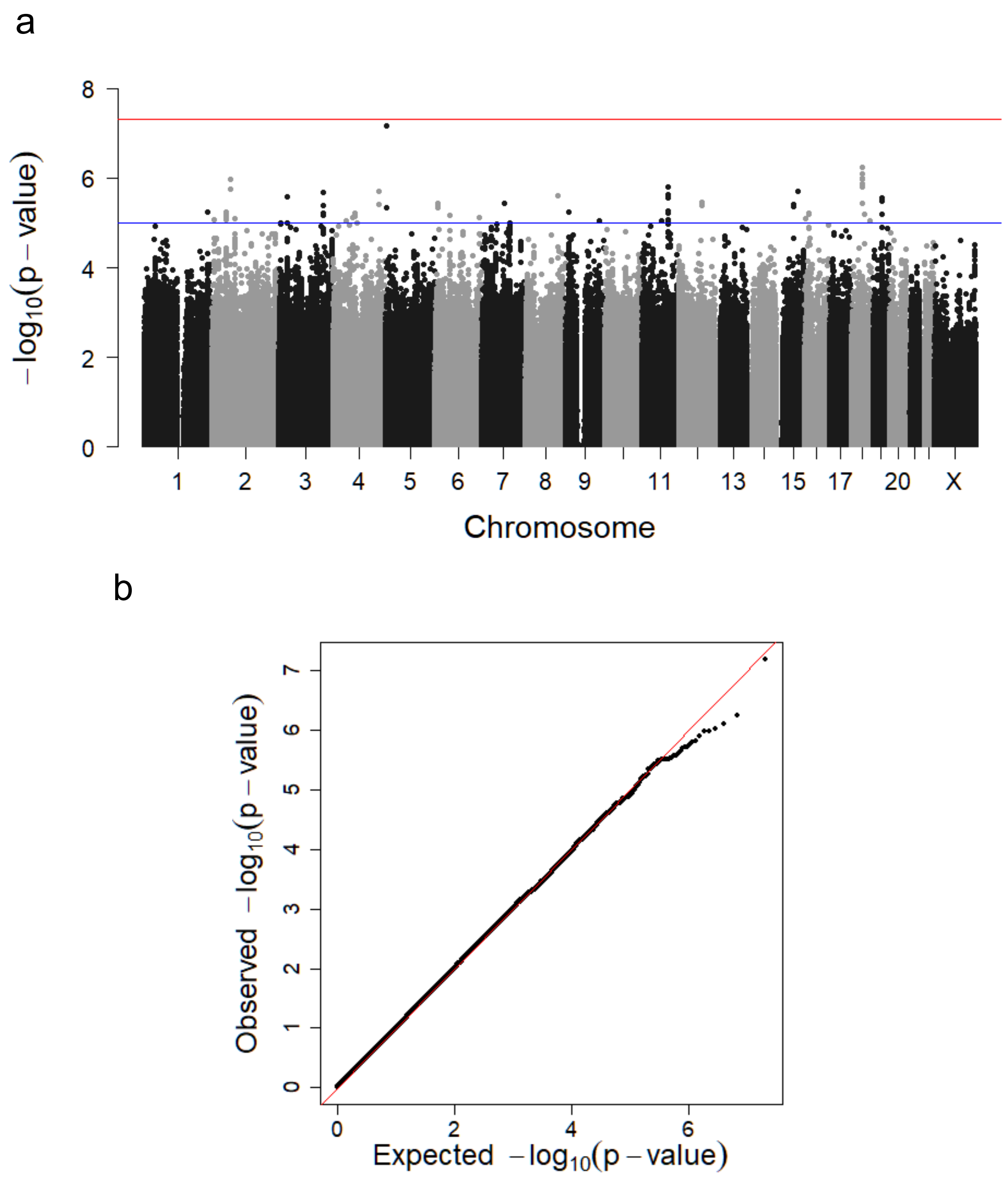

Supplementary Figure 6. A sub-analysis of the genome-wide association analysis of knee extension strength for 1,607 participants of Set 3 aged 60 years or older

(a) Manhattan plot. (b) Q-Q plot for the analysis. The genomic inflation factor (λ_{GC}) was 1.00.

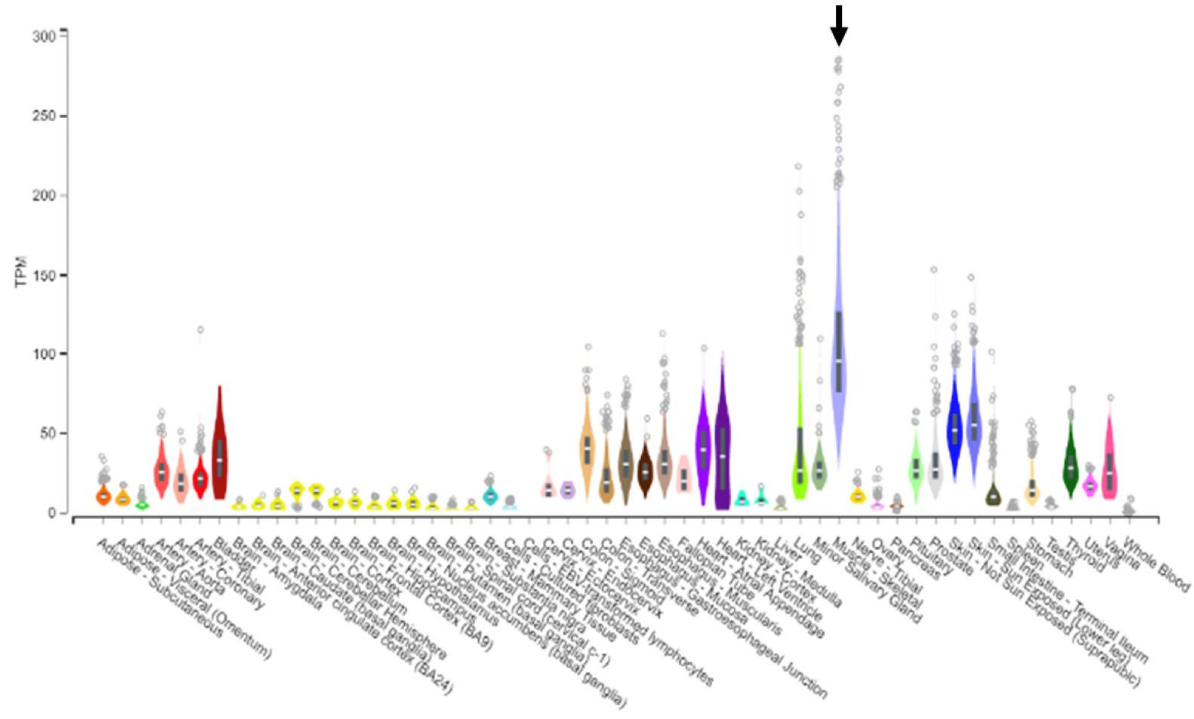

Supplementary Figure 7. The mRNA expression of *TACC2* in Gene-Tissue expression version 8
TACC2 is highly expressed in skeletal muscle (arrow) compared to other tissues or cells.

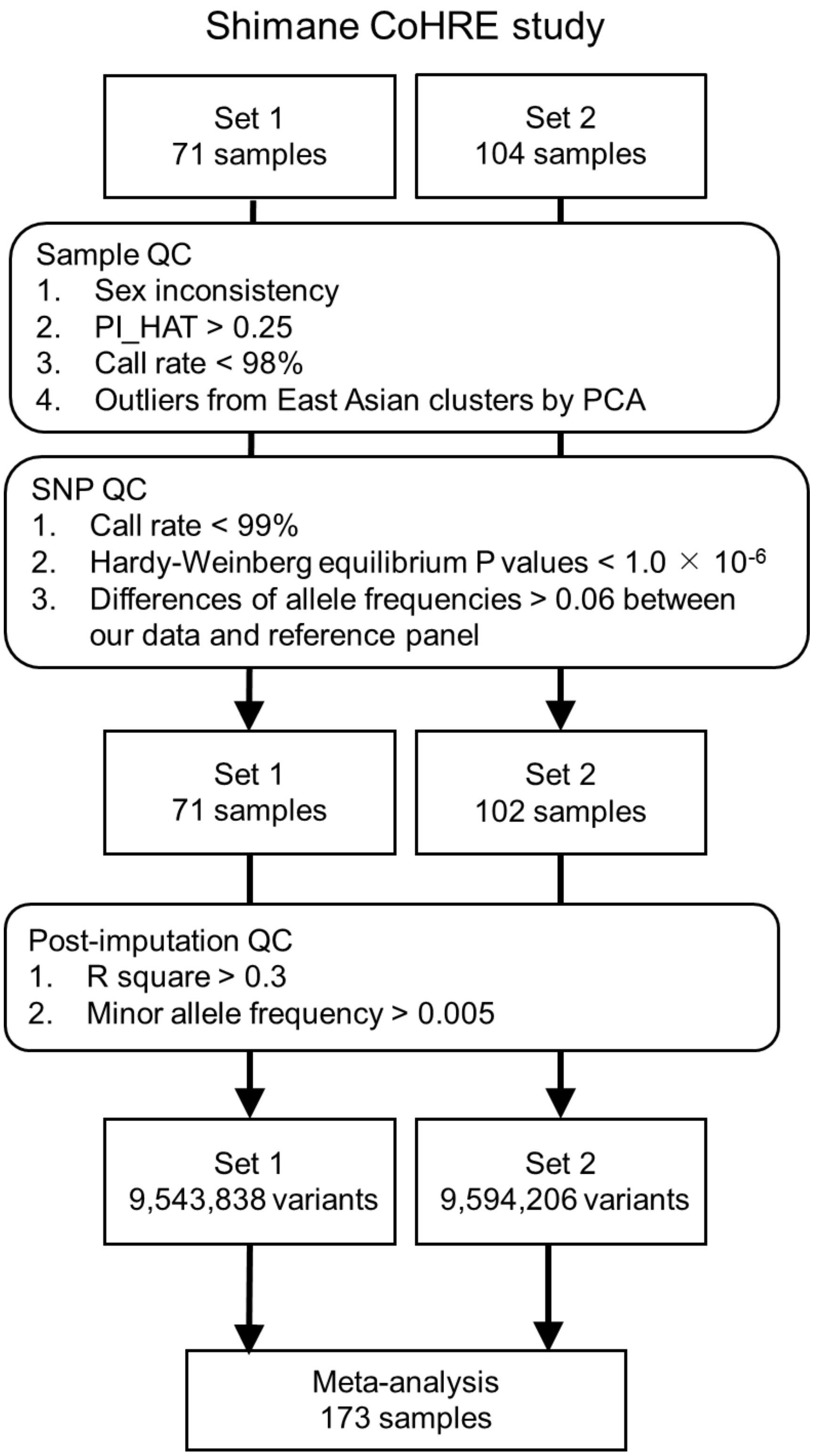

Supplementary Figure 8. Overview of the meta-analysis for the participants aged under 60 years
Two genome-wide association studies (Set 1-2) from Shimane CoHRE Study were analyzed. PCA, principal component analysis; QC, quality control.

a

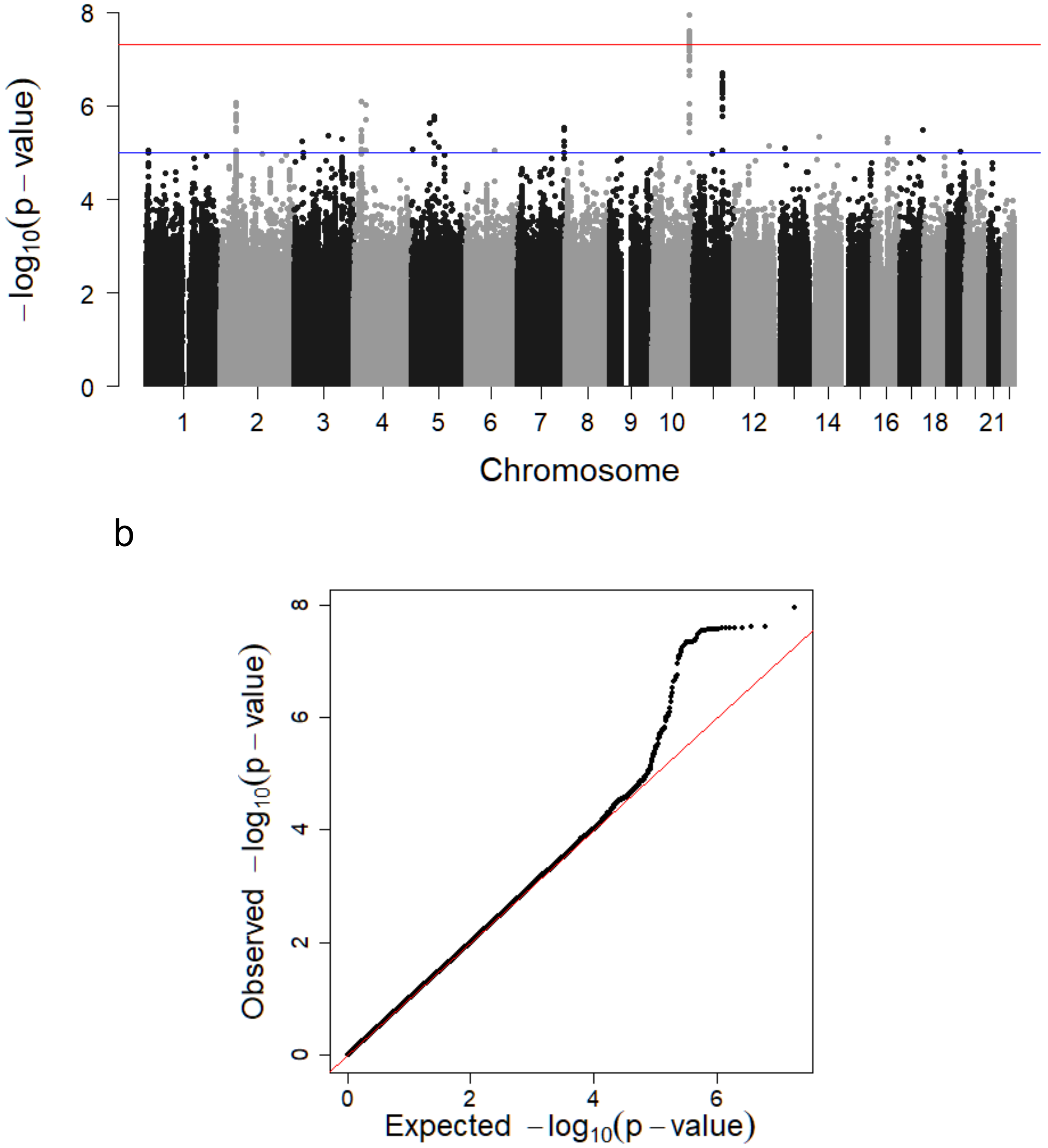

Supplementary Figure 9. A meta-analysis for 3,452 participants of Set 1-3 aged 60 years or older and 173 participants of Set 1-2 aged under 60 years

(a) Manhattan plot. A locus on chromosome 10 with genome-wide significance and many loci with suggestive genome-wide significance were identified. (b) Q-Q plot for the analysis. The genomic inflation factor (λ_{GC}) was 1.02.

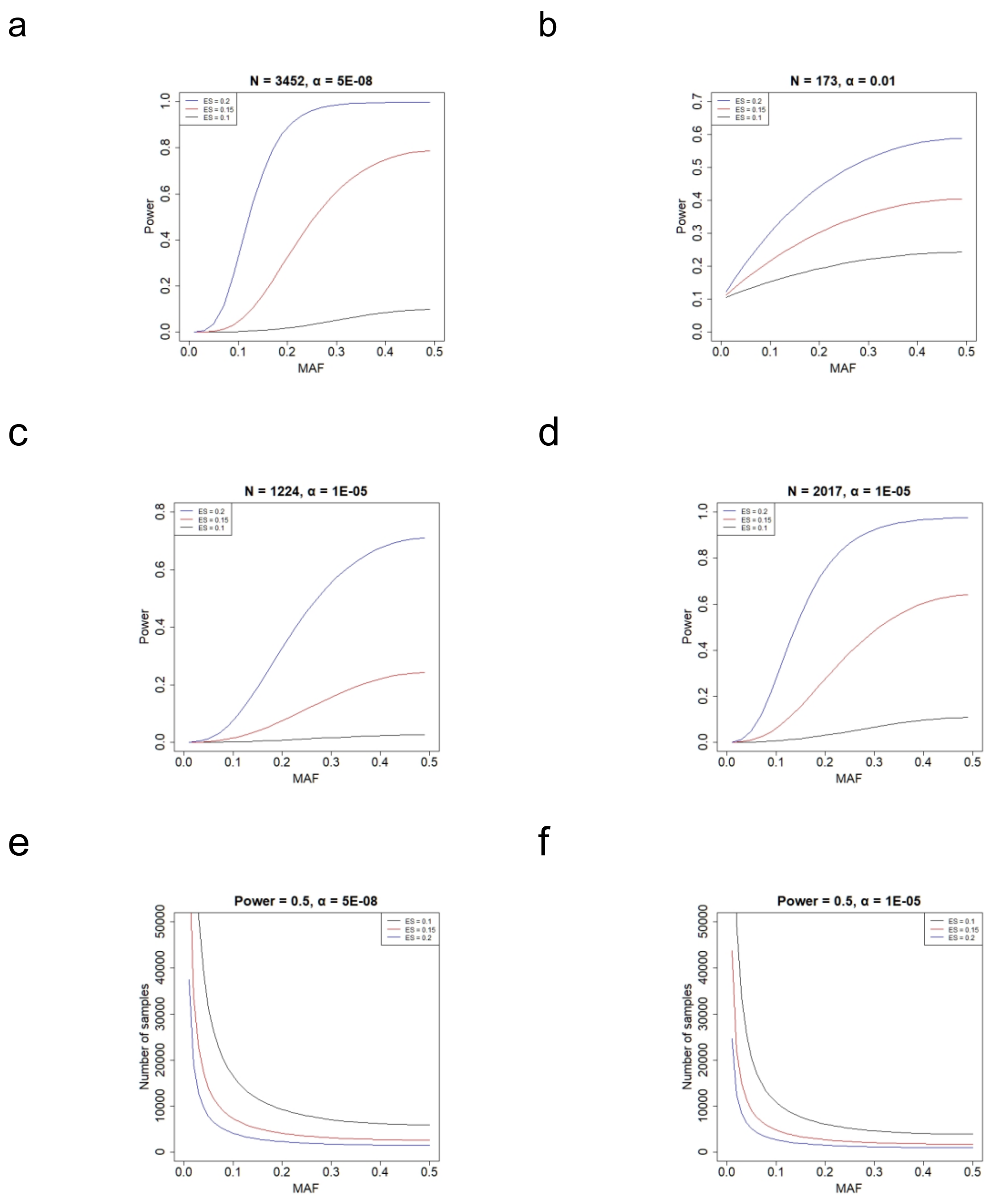

Supplementary Figure 10. Statistical power analyses

(a) The entire study (3,453 subjects) with alpha error of 5×10^{-8}. (b) 173 subjects aged under 60 years old with alpha error of 0.1. (c) 1,224 subjects of male with alpha error of 1.0×10^{-5}. (d) 2,017 subjects of female with alpha error of 1.0×10^{-5}. (e) Calculation of number of samples with alpha error of 5×10^{-8}. (f) Calculation of number of samples with alpha error of 1×10^{-5}. N, number of samples; ES, effect size; MAF, minor allele frequency.

Supplementary Table 1. A Statistical fine-mapping analysis for a significant locus

Rank	Chr	Position	PP
1	10	123810832	0.0794748
2	10	123815002	0.0417609
3	10	123814472	0.0409503
4	10	123820734	0.0345648
5	10	123820715	0.0267624
6	10	123820380	0.0267624
7	10	123819715	0.0263035
8	10	123819539	0.0263035
9	10	123817856	0.0261585
10	10	123818270	0.0261585
11	10	123818723	0.025646
12	10	123818647	0.0252792
13	10	123819352	0.0252792
14	10	123818632	0.0252792
15	10	123819748	0.0252364
16	10	123819631	0.0252364
17	10	123819795	0.0252364
18	10	123818144	0.0251438
19	10	123820363	0.024748
20	10	123817331	0.0241699
21	10	123818556	0.0238004
22	10	123818827	0.0237155
23	10	123818578	0.0233528
24	10	123819808	0.0233528
25	10	123817724	0.023235
26	10	123819480	0.0228954
27	10	123818562	0.0228903
28	10	123818851	0.021578
29	10	123821975	0.0213919
30	10	123817654	0.0206757
31	10	123817685	0.0206757
32	10	123818590	0.0199427
33	10	123818593	0.0199427
34	10	123822933	0.0186963
35	10	123822972	0.0169883
36	10	123825792	0.0143098
37	10	123825788	0.0143098

Chr, chromosome; PP, posterior probability

Supplementary Table 2. Sub-analysis in male participants aged 60 years or older

rsID	Chr	Position	Gene	Location	Ref	Alt	META			Set 1		Set 2		Set 3	
							Freq	Beta	P-value	Beta	P-value	Beta	P-value	Beta	P-value
rs141279361	1	202923428	*ADIPOR1*	5'UTR	C	T	0.022	-0.214	1.29E-01	-0.145	6.78E-01	0.112	6.50E-01	-0.449	2.38E-02
rs77607073	2	49531177	*FSHR/NRXN1*	intergenic	A	G	0.117	0.189	2.32E-03	0.213	9.32E-02	0.300	1.96E-02	0.128	1.35E-01
rs147964289	3	3825426	*CRBN/LRRN1*	intergenic	A	C	0.013	-0.519	4.58E-03	-0.901	4.48E-03	-0.425	2.59E-01	-0.266	3.45E-01
rs141616911	3	105393996	*CBLB*	intronic	T	C	0.008	0.589	1.73E-02	1.029	1.79E-01	0.086	8.64E-01	0.705	2.16E-02
rs138068168	3	158936130	*IQCJ,IQCJ-SCHIP1*	intronic	T	TTG	0.487	-0.089	4.06E-02	-0.179	3.53E-02	0.075	4.18E-01	-0.113	6.15E-02
rs11942832	4	24951385	*CCDC149*	intronic	T	C	0.320	-0.157	4.85E-04	-0.258	3.55E-03	-0.115	2.37E-01	-0.124	4.82E-02
rs118050709	4	39178700	*KLHL5/WDR19*	intergenic	C	A	0.046	-0.270	7.75E-03	-0.145	5.79E-01	-0.382	5.99E-02	-0.255	5.28E-02
rs1289351462	5	60624566	*LINC02057/ZSWIM6*	intergenic	A	ACAATGGCTTAGG	0.006	-0.470	2.55E-01	2.757	4.53E-01	0.660	5.22E-01	-0.740	1.04E-01
rs2243036	5	76122388	*F2RL1*	intronic	G	A	0.252	0.093	5.72E-02	0.144	1.14E-01	-0.008	9.38E-01	0.103	1.30E-01
rs61093400	5	108415405	*FER*	intronic	A	G	0.029	0.384	2.73E-03	0.231	6.14E-01	0.325	1.25E-01	0.445	1.00E-02
rs10749438	10	123810832	*TACC2*	intronic	G	A	0.707	-0.131	3.67E-03	-0.161	6.96E-02	-0.109	2.53E-01	-0.125	4.64E-02
rs6483495	11	96036188	*MAML2*	intronic	G	A	0.303	-0.157	2.61E-04	-0.124	1.81E-01	-0.177	4.67E-02	-0.161	5.61E-03
rs182016826	12	116219902	*TBX3/MED13L*	intergenic	T	C	0.015	-0.213	2.49E-01	-0.087	8.56E-01	-0.603	1.60E-01	-0.132	5.59E-01
rs117436582	13	37367772	*SERTM1/RFXAP*	intergenic	A	G	0.046	-0.304	3.51E-03	-0.315	8.39E-02	-0.317	1.72E-01	-0.291	5.66E-02
rs76373752	14	34645522	*EGLN3/SPTSSA*	intergenic	T	C	0.039	0.347	1.82E-03	0.439	3.50E-02	0.377	1.05E-01	0.277	8.44E-02
rs3743680	16	69153608	*CHTF8*	3'UTR	C	T	0.025	-0.171	2.58E-01	0.376	1.80E-01	-0.501	1.45E-01	-0.356	9.09E-02
rs148814682	18	69057390	*GTSCR1/LINC01541*	intergenic	C	T	0.036	0.370	7.86E-04	0.095	6.66E-01	0.187	5.33E-01	0.524	2.14E-04
rs1718047	X	32179025	*DMD*	intronic	C	A	0.858	0.135	1.24E-03	0.152	3.13E-02	0.088	3.22E-01	0.145	2.29E-02

Chr, chromosome; Ref, reference allele; Alt, alternative allele; META, meta-analysis; Freq, allele frequency of an alternative allele; Beta, beta of an alternative allele; UTR, untranslated region

Supplementary Table 3. Sub-analysis in female participants aged 60 years or older

rsID	Chr	Position	Gene	Location	Ref	Alt	META			Set 1		Set 2		Set 3	
							Freq	Beta	P-value	Beta	P-value	Beta	P-value	Beta	P-value
rs141279361	1	202923428	*ADIPOR1*	5'UTR	C	T	0.026	-0.415	4.63E-05	-0.506	3.97E-03	-0.510	2.14E-02	-0.301	4.80E-02
rs77607073	2	49531177	*FSHR/NRXN1*	intergenic	A	G	0.113	0.160	1.71E-03	0.048	6.15E-01	0.193	4.60E-02	0.213	5.97E-03
rs147964289	3	3825426	*CRBN/LRRN1*	intergenic	A	C	0.010	-0.618	2.56E-04	-0.501	8.82E-02	-0.706	4.40E-02	-0.660	1.03E-02
rs141616911	3	105393996	*CBLB*	intronic	T	C	0.007	0.759	1.70E-04	0.937	1.82E-02	1.303	8.92E-03	0.522	5.05E-02
rs138068168	3	158936130	*IQCJ,IQCJ-SCHIP1*	intronic	T	TTG	0.470	-0.133	7.30E-05	-0.179	3.08E-03	-0.111	9.46E-02	-0.113	2.65E-02
rs11942832	4	24951385	*CCDC149*	intronic	T	C	0.306	-0.127	4.11E-04	-0.041	5.23E-01	-0.185	1.31E-02	-0.157	3.53E-03
rs118050709	4	39178700	*KLHL5/WDR19*	intergenic	C	A	0.049	-0.302	8.49E-05	-0.385	6.15E-03	-0.221	1.53E-01	-0.292	1.11E-02
rs1289351462	5	60624566	*LINC02057/ZSWIM6*	intergenic	A	ACAATGGCTTAGG	0.008	-1.189	4.28E-07	-1.409	5.04E-04	-0.879	7.52E-02	-1.178	1.03E-03
rs2243036	5	76122388	*F2RL1*	intronic	G	A	0.245	0.164	2.85E-05	0.071	2.79E-01	0.236	3.64E-03	0.201	9.54E-04
rs61093400	5	108415405	*FER*	intronic	A	G	0.027	0.300	5.26E-03	0.140	5.96E-01	0.373	1.92E-02	0.281	1.09E-01
rs10749438	10	123810832	*TACC2*	intronic	G	A	0.705	-0.164	3.14E-06	-0.148	2.19E-02	-0.143	4.16E-02	-0.187	4.02E-04
rs6483495	11	96036188	*MAML2*	intronic	G	A	0.316	-0.136	5.25E-05	-0.085	1.66E-01	-0.084	2.36E-01	-0.193	8.38E-05
rs182016826	12	116219902	*TBX3/MED13L*	intergenic	T	C	0.018	-0.609	8.09E-07	-0.939	1.81E-06	-0.220	3.51E-01	-0.530	1.49E-02
rs117436582	13	37367772	*SERTM1/RFXAP*	intergenic	A	G	0.050	-0.282	2.76E-04	-0.198	9.85E-02	-0.357	5.33E-02	-0.335	6.02E-03
rs76373752	14	34645522	*EGLN3/SPTSSA*	intergenic	T	C	0.042	0.309	5.56E-04	0.355	2.60E-02	0.186	2.64E-01	0.363	1.12E-02
rs3743680	16	69153608	*CHTF8*	3'UTR	C	T	0.028	-0.432	1.48E-04	-0.751	5.65E-05	-0.453	6.27E-02	-0.121	5.01E-01
rs148814682	18	69057390	*GTSCR1/LINC01541*	intergenic	C	T	0.036	0.203	1.69E-02	0.358	1.69E-02	0.423	2.93E-02	0.012	9.21E-01
rs1718047	X	32179025	*DMD*	intronic	C	A	0.843	0.137	7.20E-04	0.272	3.03E-04	0.045	5.97E-01	0.099	8.80E-02

Chr, chromosome; Ref, reference allele; Alt, alternative allele; META, meta-analysis; Frequency, allele frequency of an alternative allele; Beta, beta of an alternative allele; UTR, untranslated region

Supplementary Table 4. Sub-analysis in the participants aged 75 years or older

rsID	Chr	Position	Gene	Location	Ref	Alt	META			Set 1		Set 2		Set 3	
							Freq	Beta	P-value	Beta	P-value	Beta	P-value	Beta	P-value
rs141279361	1	202923428	*ADIPOR1*	5'UTR	C	T	0.030	-0.230	5.55E-02	-0.167	4.58E-01	-0.039	8.71E-01	-0.369	3.59E-02
rs77607073	2	49531177	*FSHR/NRXN1*	intergenic	A	G	0.113	0.265	2.04E-05	0.161	1.53E-01	0.507	1.92E-04	0.222	1.36E-02
rs147964289	3	3825426	*CRBN/LRRN1*	intergenic	A	C	0.009	-0.398	9.63E-02	-0.132	7.45E-01	-0.888	1.99E-01	-0.462	1.60E-01
rs141616911	3	105393996	*CBLB*	intronic	T	C	0.008	0.548	2.87E-02	0.974	3.29E-02	-0.095	8.81E-01	0.496	1.46E-01
rs138068168	3	158936130	*IQCJ,IQCJ-SCHIP1*	intronic	T	TTG	0.471	-0.126	2.57E-03	-0.213	4.24E-03	-0.069	4.71E-01	-0.092	1.26E-01
rs11942832	4	24951385	*CCDC149*	intronic	T	C	0.302	-0.194	2.13E-05	-0.127	1.09E-01	-0.129	2.19E-01	-0.268	6.03E-05
rs118050709	4	39178700	*KLHL5/WDR19*	intergenic	C	A	0.046	-0.321	1.10E-03	-0.571	3.24E-03	-0.242	2.42E-01	-0.229	9.58E-02
rs1289351462	5	60624566	*LINC02057/ZSWIM6*	intergenic	A	ACAATGGCTTAGG	0.005	-1.249	3.65E-03	-1.360	5.11E-02	3.585	4.43E-01	-1.247	2.38E-02
rs2243036	5	76122388	*F2RL1*	intronic	G	A	0.244	0.073	1.32E-01	-0.006	9.40E-01	0.078	4.67E-01	0.139	6.04E-02
rs61093400	5	108415405	*FER*	intronic	A	G	0.025	0.234	9.22E-02	0.530	1.59E-01	0.310	1.32E-01	0.048	8.25E-01
rs10749438	10	123810832	*TACC2*	intronic	G	A	0.700	-0.170	7.34E-05	-0.072	3.54E-01	-0.170	7.38E-02	-0.232	1.73E-04
rs6483495	11	96036188	*MAML2*	intronic	G	A	0.316	-0.160	1.18E-04	-0.049	5.18E-01	-0.200	3.28E-02	-0.210	3.51E-04
rs182016826	12	116219902	*TBX3/MED13L*	intergenic	T	C	0.013	-0.601	1.18E-03	-0.589	1.28E-01	-0.935	1.16E-02	-0.442	8.65E-02
rs117436582	13	37367772	*SERTM1/RFXAP*	intergenic	A	G	0.043	-0.101	3.20E-01	0.008	9.60E-01	0.087	7.50E-01	-0.272	8.01E-02
rs76373752	14	34645522	*EGLN3/SPTSSA*	intergenic	T	C	0.041	0.201	7.12E-02	0.280	1.46E-01	0.118	5.71E-01	0.195	2.85E-01
rs3743680	16	69153608	*CHTF8*	3'UTR	C	T	0.027	-0.399	5.01E-03	-0.583	1.33E-02	-0.758	2.40E-02	-0.104	6.24E-01
rs148814682	18	69057390	*GTSCR1/LINC01541*	intergenic	C	T	0.039	0.244	1.75E-02	0.247	1.43E-01	0.539	4.23E-02	0.148	3.18E-01

Chr, chromosome; Ref, reference allele; Alt, alternative allele; META, meta-analysis; Freq, allele frequency of an alternative allele; Beta, beta of an alternative allele

UTR, untranslated region

Supplementary Table 5. Characteristics of the subjects aged under 60 years

	Set 1	Set 2
**Source**	Shimane CoHRE Study (1st cohort)	Shimane CoHRE Study (2nd cohort)
**Nunmber of samples**	71	102
**Sex (male/female)**	24/47	32/71
**Mean age (s.d.)**	54.6 (4.6)	51.8 (7.4)
**Mode of knee extension strength**	isometric	isometric
**Device**	QTM-05F	QTM-05F
**Mean muscle strength/ Body weight [kg/kg] (s.d.)**	0.776 (0.260)	0.645 (0.175)
**Genotyping platform**	Ilumina, OmniExpressExome	Ilumina, Asian Screening Array

"Number of samples" is after quality control. s.d., standard deviation

Supplementary Table 6. Sub-analysis in the participants aged under 60 years

rsID	Chr	Position	Gene	Location	Ref	Alt	META			Set 1		Set 2	
							Freq	Beta	P-value	Beta	P-value	Beta	P-value
rs141279361	1	202923428	*ADIPOR1*	5'UTR	C	T	0.029	-0.051	8.65E-01	-0.020	9.61E-01	-0.088	8.43E-01
rs77607073	2	49531177	*FSHR/NRXN1*	intergenic	A	G	0.109	0.335	6.65E-02	0.201	4.52E-01	0.455	7.35E-02
rs147964289	3	3825426	*CRBN/LRRN1*	intergenic	A	C	0.019	0.068	8.76E-01	0.102	8.61E-01	0.025	9.69E-01
rs141616911	3	105393996	*CBLB*	intronic	T	C	0.012	-0.018	9.71E-01	-0.404	5.65E-01	0.390	5.88E-01
rs138068168	3	158936130	*IQCJ,IQCJ-SCHIP1*	intronic	T	TTG	0.458	-0.110	3.25E-01	-0.063	7.13E-01	-0.143	3.30E-01
rs11942832	4	24951385	*CCDC149*	intronic	T	C	0.299	-0.093	4.53E-01	-0.213	2.60E-01	-0.001	9.96E-01
rs118050709	4	39178700	*KLHL5/WDR19*	intergenic	C	A	0.062	-0.138	5.30E-01	-0.928	7.26E-03	0.457	1.19E-01
rs1289351462	5	60624566	*LINC02057/ZSWIM6*	intergenic	A	ACAATGGCTTAGG	0.002	2.140	8.27E-01	21.778	9.44E-02	-25.309	9.85E-02
rs2243036	5	76122388	*F2RL1*	intronic	G	A	0.247	0.219	7.86E-02	0.320	6.85E-02	0.110	5.43E-01
rs61093400	5	108415405	*FER*	intronic	A	G	0.037	-0.099	7.56E-01	-0.138	8.10E-01	-0.082	8.33E-01
rs10749438	10	123810832	*TACC2*	intronic	G	A	0.687	-0.200	1.05E-01	-0.316	9.42E-02	-0.110	5.06E-01
rs6483495	11	96036188	*MAML2*	intronic	G	A	0.305	0.063	5.87E-01	0.045	7.86E-01	0.081	6.22E-01
rs182016826	12	116219902	*TBX3/MED13L*	intergenic	T	C	0.023	-0.110	7.79E-01	-7.786	8.24E-01	-0.109	7.82E-01
rs117436582	13	37367772	*SERTM1/RFXAP*	intergenic	A	G	0.040	-0.107	7.23E-01	-0.533	1.72E-01	0.548	2.55E-01
rs76373752	14	34645522	*EGLN3/PTSSA*	intergenic	T	C	0.044	0.193	4.99E-01	0.109	7.83E-01	0.287	4.91E-01
rs3743680	16	69153608	*CHTF8*	3'UTR	C	T	0.021	0.124	7.78E-01	-0.434	4.86E-01	0.687	2.73E-01
rs148814682	18	69057390	*GTSCR1/INC01541*	intergenic	C	T	0.029	-0.278	3.87E-01	-0.513	3.03E-01	-0.106	8.02E-01

Chr, chromosome; Ref, reference allele; Alt, alternative allele; META, meta-analysis; Freq, allele frequency of an alternative allele; Beta, beta of an alternative allele

UTR, untranslated region

Supplementary note

We investigated if suggestive variants identified in the current study are associated with sarcopenia-related traits such as lean body mass, frailty, walking pace fatigue, testosterone and IGF1 in the UK Biobank. A suggestive variant, rs13022242 is nominally associated with lean body mass in the same direction. A suggestive variant, rs138068168, is nominally associated with testosterone in the same direction. rs13314849, which is in linkage disequilibrium with rs138068168, is nominally associated with frailty in the same direction. A suggestive variant, rs2243036 is also nominally associated with testosterone in the same direction. A suggestive variant, rs118050709 is nominally associated with IGF1 in the same direction.
