# Supplementary material for: A genome-wide association study identifies a locus associated with knee extension strength in older Japanese individuals
Source: Commun Biol. 2024 May 20;7:513. doi: 10.1038/s42003-024-06108-6 (PMC11106293; doi:10.1038/s42003-024-06108-6)
Supplement: Supplementary file 3 — Description of Additional Supplementary Files [file 42003_2024_6108_MOESM3_ESM.docx]

**Description of Additional Supplementary Files**

**File name:** Supplementary data 1

**Description:** All the source data behind the graphs in the paper
