# Supplementary material for: A genome-wide association study identifies a locus associated with knee extension strength in older Japanese individuals
Source: Commun Biol. 2024 May 20;7:513. doi: 10.1038/s42003-024-06108-6 (PMC11106293; doi:10.1038/s42003-024-06108-6)
Supplement: Supplementary file 5 — Reporting Summary [file 42003_2024_6108_MOESM5_ESM.pdf]

nature portfolio

Corresponding author(s): Chikashi Terao
Last updated by author(s): Feb 16, 2024

Reporting Summary

Nature Portfolio wishes to improve the reproducibility of the work that we publish. This form provides structure for consistency and transparency in reporting. For further information on Nature Portfolio policies, see our Editorial Policies and the Editorial Policy Checklist.

Statistics

For all statistical analyses, confirm that the following items are present in the figure legend, table legend, main text, or Methods section.

n/a	Confirmed	
☐	☒	The exact sample size (*n*) for each experimental group/condition, given as a discrete number and unit of measurement
☐	☒	A statement on whether measurements were taken from distinct samples or whether the same sample was measured repeatedly
☐	☒	The statistical test(s) used AND whether they are one- or two-sided<br>*Only common tests should be described solely by name; describe more complex techniques in the Methods section.*
☐	☒	A description of all covariates tested
☐	☒	A description of any assumptions or corrections, such as tests of normality and adjustment for multiple comparisons
☐	☒	A full description of the statistical parameters including central tendency (e.g. means) or other basic estimates (e.g. regression coefficient) AND variation (e.g. standard deviation) or associated estimates of uncertainty (e.g. confidence intervals)
☐	☒	For null hypothesis testing, the test statistic (e.g. *F*, *t*, *r*) with confidence intervals, effect sizes, degrees of freedom and *P* value noted<br>*Give P values as exact values whenever suitable.*
☐	☒	For Bayesian analysis, information on the choice of priors and Markov chain Monte Carlo settings
☐	☒	For hierarchical and complex designs, identification of the appropriate level for tests and full reporting of outcomes
☐	☒	Estimates of effect sizes (e.g. Cohen's *d*, Pearson's *r*), indicating how they were calculated

Our web collection on statistics for biologists contains articles on many of the points above.

Software and code

Policy information about availability of computer code

Data collection	All participants were collected in Shimane CoHRE Study and Bunkyo Health Study. Shimane CoHRE Study is the cross-sectional study which is an ongoing health examination for the community-dwelling people in Shimane prefecture, Japan. Bunkyo Health Study is a prospective cohort study of over 10 years, which recruited older subjects aged 65–84 years living in Bunkyo-Ku, an urban area in Tokyo, Japan.
Data analysis	We conducted quality control (QC) with PLINK v1.90 and imputation with minimac4; We conducted a GWAS by applying a linear mixed model using fastGWA. The Meta-analysis was done with METAL (version March 2011).

For manuscripts utilizing custom algorithms or software that are central to the research but not yet described in published literature, software must be made available to editors and reviewers. We strongly encourage code deposition in a community repository (e.g. GitHub). See the Nature Portfolio guidelines for submitting code & software for further information.

Data

Policy information about availability of data

All manuscripts must include a data availability statement. This statement should provide the following information, where applicable:

- Accession codes, unique identifiers, or web links for publicly available datasets
- A description of any restrictions on data availability
- For clinical datasets or third party data, please ensure that the statement adheres to our policy

The GWAS summary statistics will be available at http://jenger.riken.jp/result.

Human research participants

Policy information about studies involving human research participants and Sex and Gender in Research.

Reporting on sex and gender	In the current study, we confirmed the consistency between self-reported sex and genotype. We excluded the samples with sex inconsistency between clinical data and genotype.
Population characteristics	The individuals in the GWAS were all Japanese.
Recruitment	We collected samples in Shimane CoHRE Study and Bunkyo Health Study.
Ethics oversight	The study was approved by the Ethics Committee of Shimane University School of Medicine and Juntendo University.

Note that full information on the approval of the study protocol must also be provided in the manuscript.

Field-specific reporting

Please select the one below that is the best fit for your research. If you are not sure, read the appropriate sections before making your selection.

☒ Life sciences ☐ Behavioural & social sciences ☐ Ecological, evolutionary & environmental sciences

For a reference copy of the document with all sections, see nature.com/documents/nr-reporting-summary-flat.pdf

Life sciences study design

All studies must disclose on these points even when the disclosure is negative.

Sample size	We conducted three GWASes in a total of 3,452 Japanese aged 60 years or older. We did not perform any statistical methods to determine the sample size.
Data exclusions	For quality control of samples, we excluded those with: (1) a sample call rate of <0.98; and (2) outliers from East Asian clusters identified by a principal component analysis using genotyped samples. For quality control of genotypes, we excluded variants meeting any of the following criteria: (1) call rate < 99%; (2) P value for Hardy-Weinberg equilibrium (HWE) < 1.0 × 10-6. We also excluded SNP with a large allele frequency difference between the reference panel and the samples (> 0.06).
Replication	We could not reprocude the study because we conducted GWASes using all samples we have.
Randomization	Not applicable as we utilized all samples.
Blinding	All individual data was anonymized to protect privacy.

Reporting for specific materials, systems and methods

We require information from authors about some types of materials, experimental systems and methods used in many studies. Here, indicate whether each material, system or method listed is relevant to your study. If you are not sure if a list item applies to your research, read the appropriate section before selecting a response.

Materials & experimental systems

n/a	Involved in the study	
☒	☐	Antibodies
☒	☐	Eukaryotic cell lines
☒	☐	Palaeontology and archaeology
☒	☐	Animals and other organisms
☒	☐	Clinical data
☒	☐	Dual use research of concern

Methods

n/a	Involved in the study	
☒	☐	ChIP-seq
☒	☐	Flow cytometry
☒	☐	MRI-based neuroimaging
